# Impact of Whole, Fresh Fruit Consumption on Energy Intake and Adiposity: A Systematic Review

**DOI:** 10.3389/fnut.2019.00066

**Published:** 2019-05-08

**Authors:** Stephan J. Guyenet

**Affiliations:** Self-employed, Seattle, WA, United States

**Keywords:** fruit, adiposity, body weight, obesity, energy intake, sugar

## Abstract

**Background:** The energy content of whole, fresh fruit derives primarily from simple sugars, which are currently under heightened scrutiny for their potential contribution to obesity and chronic disease risk. Yet fruit also has a relatively low energy density, moderate palatability/reward value, and high fiber content, which together may limit energy intake. Although reasoned arguments can be made that fruit is fattening or slimming, the question is best resolved empirically.

**Methods:** Methods were preregistered with PROSPERO (CRD42018111830). The primary outcome is the impact of whole, fresh fruit consumption on measures of adiposity including body weight in randomized controlled trials (RCTs). Secondary outcomes are the impact of whole, fresh fruit consumption on energy intake in RCTs, and the association between whole, fresh fruit consumption and changes in measures of adiposity in prospective observational studies. CENTRAL and PubMed databases were searched through October 2018. Cochrane risk of bias tool was used to assess risk of bias in RCTs, and the GRADE method was used to judge and convey the certainty of conclusions. Reporting follows PRISMA guidelines.

**Results:** RCTs, and particularly those of higher quality, suggest that increasing whole, fresh fruit consumption promotes weight maintenance or modest weight loss over periods of 3–24 weeks (moderate certainty), with limited evidence suggesting that a high intake of fruit favors weight loss among people with overweight or obesity. Consistent with this, single-meal RCTs suggest that consuming whole, fresh fruit tends to decrease energy intake, particularly when consumed prior to a meal or when displacing more energy-dense foods (moderate certainty). Prospective observational studies suggest that habitually higher fruit intake is not associated with weight change, or is associated with modest protection against weight gain, over five or more years.

**Conclusions:** Current evidence suggests that whole, fresh fruit consumption is unlikely to contribute to excess energy intake and adiposity, but rather has little effect on these outcomes or constrains them modestly. Single-meal RCTs, RCTs lasting 3–24 weeks, and long-term observational studies are relatively consistent in supporting this conclusion. Whole, fresh fruit probably does not contribute to obesity and may have a place in the prevention and management of excess adiposity.

## Introduction

### Rationale

Worldwide, the total *per capita* burden of disease continues to decline, but it does not do so uniformly. Technological and economic progress have substantially relieved the ancient burdens of starvation, infectious disease, and accidents, yet they have ushered in a new era of non-communicable disorders such as obesity, diabetes, and coronary heart disease (1, 2). A key contributor to these disorders is the overconsumption of energy and accumulation of excess adipose tissue (3–6).

Sucrose and other simple sugars with a sweet taste, henceforth “sugar,” have long been suspected as a culprit in energy overconsumption and excess adiposity. In 1980, the United States Department of Agriculture Dietary Guidelines advised the public to “avoid too much sugar” and, as part of a four-point plan for reducing weight, “eat less sugar and sweets” (7). Yet scrutiny of sugar has intensified recently, both within the scientific community and outside it, with certain researchers and popular writers arguing that sugar is a particularly potent driver of obesity and non-communicable disease risk (8–10). Observational and experimental findings indeed suggest that in sufficient quantity, refined sugar can increase energy intake and adiposity (11, 12). The energy content of sweet fruits is primarily in the form of sugar. If sugar is a particularly potent driver of obesity and non-communicable disease risk, this raises the possibility that even whole, fresh fruit may have similar effects, and that conventional advice to increase fruit consumption may be misguided.

On the other hand, fruit differs from refined sugar-containing foods in important respects, such as its lower energy density, lower palatability/reward value, higher fiber content, and higher concentration of essential and non-essential micronutrients. Some of these properties are expected to limit energy intake and adiposity, and together they may render fruit slimming relative to other commonly-eaten foods. Furthermore, the human evolutionary lineage has likely consumed substantial quantities of fruit for tens of millions of years prior to the emergence of obesity and cardiometabolic disease as common health problems, suggesting that it is unlikely to be a major contributor to these conditions (13).

Although reasoned arguments can be made that fruit is fattening or slimming, the question is best resolved empirically. Previous reviews have addressed similar topics (14–17), concluding that fruit aids in the prevention of excess energy intake, is unlikely to increase adiposity, and may reduce adiposity. However, the current review is the most recent to comprehensively review the randomized controlled trial (RCT) and prospective observational literature on whole, fresh fruit intake specifically. Further, it employs best-practice systematic review methods including detailed preregistration of methods with a well-defined search strategy, adherence to PRISMA reporting guidelines, assessment of study bias using the Cochrane risk of bias tool, and assessment of certainty of conclusions using the GRADE method.

### Objectives

The objective of this review is to systematically review the randomized controlled trial and prospective observational research literature on the impact of whole, fresh fruit consumption on energy intake and measures of adiposity, and synthesize available studies to form overall conclusions. All studies involving human subjects are considered, and RCTs must report between-group comparisons that isolate differences in whole, fresh fruit consumption as a variable.

### Research Question

What is the impact of whole, fresh fruit consumption on energy intake and adiposity?

## Methods

### Protocol and Registration

A protocol for this review was preregistered in the PROSPERO systematic review registry prior to initiating literature searches (CRD42018111830)^1^, with the exception of brief preliminary searches used to develop the search method. The primary outcome is the impact of whole, fresh fruit consumption on measures of adiposity, as measured by RCTs. A secondary outcome is the impact of whole, fresh fruit consumption on energy intake, as measured by RCTs. An additional secondary outcome is the association of whole, fresh fruit intake with changes of measures of adiposity, as measured by prospective observational studies.

### Eligibility Criteria

“Fruit” is defined in the common/culinary sense of a sweet, seed-bearing plant tissue. Fruit varieties that lack seeds are included. “Whole” and “fresh” denote fruit that has not been significantly processed, i.e., raw and whole rather than cooked, pureed, dried, juiced, or powdered. Raw fruit that has been peeled or cut into bite-sized pieces is included. “Change in adiposity” is defined as change in body weight and/or other direct or indirect measures of body fatness, including but not limited to body fat percentage, body mass index, and waist circumference. “RCT” is defined as a study that assigns subjects to different intervention conditions using randomization or pseudorandomization, such that outcomes can be compared between randomized groups. “Prospective observational study” is defined as a non-intervention study that collects data on exposure variables at an earlier time point, and outcomes at a later time point, and reports the association between the two.

Studies were required to be (1) RCTs on the impact of whole, fresh fruit consumption on measures of adiposity including body weight, (2) RCTs on the impact of whole, fresh fruit consumption on energy intake, or (3) prospective observational studies on the association between fruit consumption and body weight and/or adiposity. Only published studies were considered. No language restrictions were applied. All RCTs and prospective observational studies conducted in humans and published through October 2018 were considered.

To be eligible, RCTs must include a between-group difference in fruit intake. The experimental design must isolate between-group differences in fruit intake as a variable, without substantial concurrent between-group differences in other variables such as vegetable intake. An equivalent non-fruit intervention in a comparison group, such as increased nut intake, is permitted. Energy intake of diets must not be strictly controlled to permit differences in energy intake and adiposity to arise. RCTs must report differences in measures of adiposity, or energy intake, between groups, or such differences must be calculable from data available in the manuscript. Changes in weight and/or adiposity must be measured with a minimum of 2 weeks between baseline and end line to allow meaningful differences in adiposity to emerge, while energy intake RCTs can represent any duration.

For observational outcomes, studies must be prospective observational studies that report the association between the consumption of fresh, whole fruit consumption and subsequent changes in measures of adiposity including body weight. During the course of study selection, the author felt it was appropriate to add one exclusion criterion that was not prespecified: observational studies were excluded if they were potentially confounded by a concurrent intervention. For example, an observational study that reports the association between fruit intake and weight change may be confounded if it is conducted in subjects that received advice to increase fruit intake as part of a weight loss intervention. This criterion excluded four studies, whose findings are broadly similar to those that met inclusion criteria (18–21).

### Search Strategy

The search strategy was designed in collaboration with Ben Harnke, Education and Reference Librarian, the University of Colorado Health Sciences Library. RCT searches were conducted in the Cochrane controlled register of trials (CENTRAL), and prospective observational study searches were conducted in PubMed. CENTRAL compiles RCTs from multiple sources including PubMed, Embase, Clinicaltrials.gov, handsearches, and other biomedical resources. Brief preliminary searches were used to develop the search method by verifying that studies identified in previous review papers were present (14–16). Formal searches were conducted in November 2018, following preregistration.

The adiposity RCT search employed the following search terms in CENTRAL: ((Fruit OR fruits):ti,ab OR [mh Fruit]) AND ((weight OR “body mass index” OR BMI OR “waist circumference” OR “body fat” OR adiposity OR overweight OR obes^*^ OR leanness OR Overnutrition OR “over nutrition”):ti,ab OR [mh ”Body Weight”] OR [mh “Body Weight Changes”] OR [mh “Overweight”] OR [mh “Thinness”] OR [mh “Body Fat Distribution”]).

The energy intake RCT search employed the following search terms in CENTRAL: ((Fruit OR fruits):ti,ab OR [mh Fruit]) AND ((“energy” OR calorie^*^ OR caloric OR kilocalorie^*^ OR kcal OR “joule” OR kilojoule OR kJ):tiab) AND ((intake OR consum^*^ OR ingest^*^ OR ate OR eat OR eating):ti,ab OR [mh “Energy Intake”] OR [mh Eating]).

The prospective observational study search employed the following search terms in PubMed, in addition to the “observational study” publication type filter: (Fruit[tiab] OR fruits[tiab] OR “Fruit”[Mesh:NoExp]) AND (weight[tiab] OR “body mass index”[tiab] OR BMI[tiab] OR “waist circumference”[tiab] OR “body fat”[tiab] OR adiposity[tiab] OR overweight[tiab] OR obes^*^[tiab] OR leanness[tiab] OR Overnutrition[tiab] OR “over nutrition”[tiab] OR “Body Weight”[Mesh:NoExp] OR “Body Weight Changes”[Mesh] OR “Overweight”[Mesh] OR “Thinness”[Mesh] OR “Body Fat Distribution”[Mesh]) AND (prospective^*^[tiab] OR cohort[tiab] OR longitudinal[tiab] OR follow up stud^*^[tiab] OR followup stud^*^[tiab] OR incidence stud^*^[tiab] OR “Cohort Studies”[Mesh]).

In addition, previous review papers on fruit, energy intake, and adiposity were hand searched for relevant studies (14–16).

### Study Selection

After search records were identified, SG examined titles and abstracts for studies that met inclusion criteria. Potential candidates were compiled in Excel spreadsheets for examination of full text. SG then examined the full text of each study to verify that inclusion criteria were satisfied, resulting in the exclusion of some studies.

### Data Collection

Data were extracted from studies that met inclusion criteria into Excel spreadsheets and Cochrane Review Manager 5.3. Data presented in manuscripts were taken at face value and authors were not contacted for additional information. For RCTs, the following data were extracted: first author, year of publication, number and characteristics of subjects, summary of intervention, duration of intervention, between-group difference in weight with statistical significance, between-group difference in other measures of adiposity with statistical significance, Cochrane risk of bias assessment (based on random sequence generation, allocation concealment, blinding of participants and personnel, blinding of outcome assessment, incomplete outcome data, selective reporting), additional noteworthy study features. When not directly reported, between-group differences in measures of adiposity and energy intake were calculated based on available data whenever possible.

For observational studies, the following data were extracted: first author, year, number and characteristics of subjects, measure of fruit intake, length of follow-up, covariates accounted for, association between fruit consumption and weight change with statistical significance, association between fruit consumption and other measures of adiposity with statistical significance, additional noteworthy study features. Data from the most adjusted model that does not include energy intake as a covariate were generally preferred, since energy intake is a mechanism by which fruit intake may impact body weight. If an unadjusted or minimally adjusted model was the only one available without energy intake as a covariate, the most adjusted model was selected.

Body mass index measures were only collected if body weight was not reported, since the two measures are redundant in adults who have achieved their final height.

### Risk of Bias

Risk of bas was estimated using the Cochrane risk of bias tool for individual studies, according to Cochrane guidelines^2^ and using Cochrane Review Manager 5.3, and GRADE was used to judge and communicate the certainty of conclusions for each outcome^3^ Risk of bias score was used as a criterion to weight the informativeness of each study when synthesizing conclusions, and GRADE was used to judge and communicate the certainty of each conclusion. Risk of bias graphs and summaries were created using Cochrane Review Manager 5.3.

### Synthesis of Results

This review employed a narrative synthesis method whereby study quality was evaluated and conclusions were informally weighted according to study quality (informal, i.e., a quantitative method was not applied to weight the informativeness of individual studies). Among the three outcomes considered, the preregistered primary outcome was assigned the highest weight. Reporting follows PRISMA guidelines (22). Quantitative meta-analysis of RCTs was judged to be suboptimal in this context, given the limited number of studies identified, the fact that the study pool would have been further narrowed by applying more stringent meta-analysis inclusion criteria, and widely varying study methods and quality. Although meta-analyses are commonly performed on fewer than ten studies, typical methods for accomplishing this do not adequately control the false positive rate, particularly in the context of high heterogeneity (23). Finally, given the high risk of bias of most studies identified, the author believes it is more informative to focus on high-quality trials than to pool their findings with less informative trials.

## Results

### Study Selection and Characteristics

**Figure 1:**
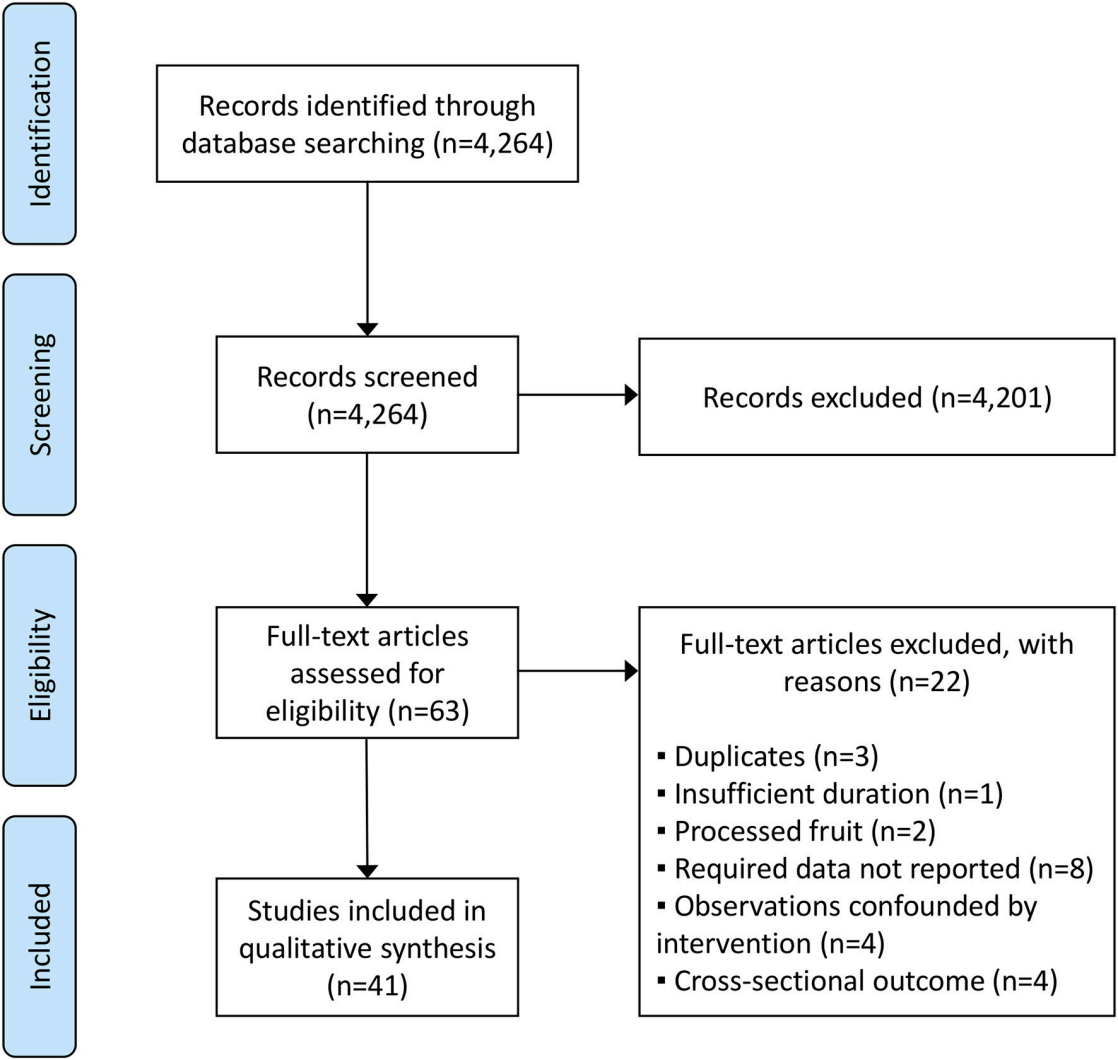
PRISMA flow diagram summarizing the study identification and selection process.

Figure 1 summarizes the search and study selection process using the PRISMA flow diagram. 4,264 records were identified in database searches, 1,671 for body weight RCTs, 848 for energy intake RCTs, and 1,745 for prospective observational studies. 4,201 records were excluded on the basis of title and abstract contents, leaving 63 potentially eligible studies; 16 body weight RCTs, 10 energy intake RCTs, and 37 observational studies. Many studies were excluded on first pass because they did not isolate whole, fresh fruit intake as a variable; for example, they reported associations between combined fruit/vegetable intake and weight outcomes. Upon inspection of full-text manuscripts, 41 studies met inclusion criteria; 11 body weight RCTs, 5 energy intake RCTs, and 25 observational studies. Reasons for exclusion were that studies were duplicates (*n* = 3), interventions were not long enough to meet inclusion criteria (*n* = 1), interventions involved processed rather than whole, fresh fruit (*n* = 2), studies did not report data that are pertinent to this review (*n* = 8), observational studies were potentially confounded by a concurrent intervention (*n* = 4), and observational studies reported data in a cross-sectional rather than prospective manner (*n* = 4).

RCTs reporting the impact of whole, fresh fruit consumption on body weight and adiposity were published between 1992 and 2016 and are summarized in Table 1. Of the 11 RCTs identified, two included fewer than 20 subjects, four included 21–50 subjects, two included 51–100 subjects, and three included more than 100 subjects. Interventions represented a variety of fruit types, but guava (*n* = 3), apple (*n* = 2), and grapefruit (*n* = 2) were named more commonly than other fruit. The quantity of fruit varied widely, with the highest intake representing a goal of 15 percent of energy intake from fruit-derived fructose, which implies ~30 percent of total energy intake from fruit. Interventions lasted 3–24 weeks, with 6 and 12 weeks being most common. All RCTs reported weight changes, and six also reported changes in other measures of adiposity. Four trials reported performing a power calculation as part of trial design, and three trials were preregistered, of which two were preregistered for an adiposity-related outcome.

**Table 1:** Primary outcome: impact of whole, fresh fruit consumption on measures of adiposity in RCTs.

**Trial**	**Subjects**	**Intervention**	**Duration**	**Weight difference**	**Adiposity difference**	**Notes**
Singh et al. (24)	Men and women with essential hypertension. 131 randomized; 66 guava, 65 control.	0.5–1.0 kg of fresh guava fruit per day before meals vs. no intervention	12 weeks	−0.4 kg (*p* = NS)	Not reported	No power calculation. Not preregistered. Subjects were asked to maintain weight.
Singh et al. (25)	Men and women with essential hypertension and mild hypercholesterolemia. 101 randomized; 52 guava; 49 control.	0.5–1.0 kg of fresh guava fruit per day before meals vs. no intervention	24 weeks	−0.1 kg (*p* = NS)	Not reported	No power calculation. Not preregistered.
de Oliveira et al. (26)	Hypercholesterolemic, overweight, non-smoking women, 30–50 years of age. 49 randomized; number in each group unclear.	3 apples, pears, or oat cookies per day. Hypocaloric diet for all subjects.	12 weeks	−0.33 kg (*p* = 0.003)	Not reported	No power calculation. Not preregistered. May have pooled two fruit groups that were individually randomized.
Rodriguez et al. (27)	Women with obesity. 15 randomized; 8 low-fruit; 7 high-fruit.	Low-fruit diet vs. high-fruit diet (goal of 5 vs. 15% of kcal from fructose). Hypocaloric diet for all subjects.	8 weeks	+0.3 kg (*p* = 0.781)	−0.4% BF (*p* = 0.231); −3.1 cm WC (*p* = 0.048); 0.01 WHR (*p* = 0.395)	No power calculation. Not preregistered. Very small sample size.
Fujioka et al. (28)	Men and women with obesity. 91 randomized; 24 grapefruit capsules + apple juice; 22 placebo + apple juice; 21 placebo + grapefruit juice; 24 placebo + fresh grapefruit.	3x a day prior to meals: Group A, Grapefruit capsules + 7 oz apple juice; Group B, placebo capsules + 7 oz apple juice; Group C placebo capsules + 8 oz grapefruit juice; Group D placebo capsules + half a fresh grapefruit. 20–30 min of walking 3–4X per week for all groups.	12 weeks	−1.4 kg vs. group B (*p* = 0.048); −0.1 kg vs. group C (*p* = NS)	NS difference in % BF (impedance)	Performed a power calculation. Not preregistered.
Rush et al. (29)	Healthy men and women. 12 randomized; 6 kiwi; 6 control.	One kiwi fruit per 30 kg body weight vs. no kiwi fruit. General diet advice and a pedometer for all subjects.	3 weeks (fruit intervention)	+0.9 kg (*p* = NS)	Not reported	No power calculation. Not preregistered. Very small sample size.
Madero et al. (30)	Men and women with overweight or obesity. 131 randomized; 66 low-fructose diet; 65 natural fructose diet.	Low-fructose diet (<20 g/d) vs. moderate natural-fructose diet (50–70 g/d mostly from fruit). Hypocaloric diet for all subjects.	6 weeks	−1.36 (*p* = 0.02)	−0.8% BF (*p* = 0.1); −0.15 WHR (*p* = 0.41)	Performed a power calculation. Preregistered with anthropometric changes as primary outcome (NCT00868673).
Dow et al. (31)	Men and premenopausal women with overweight or obesity. 74 randomized; 32 control; 42 grapefruit.	Half a fresh grapefruit with each meal (3X per d) vs. no intervention. All subjects were assigned a baseline diet low in fruit and vegetables.	6 weeks	−0.5 (*p* = 0.119)	0.55% BF (*p* = 0.337, impedance); −1.22 cm WC (*p* = 0.062); 0.0 WHR (*p* = 0.470)	Performed a power calculation. Preregistered with weight change as primary outcome (NCT01452841).
Ravn-Haren et al. (32)	Healthy men and women. 34 randomized; number per group unclear.	550 g/d whole apple; 22 mg/d apple pomace; 500 mL/d cloudy apple juice; 500 mL/d clear apple juice; no intervention. Baseline diet was low in polyphenols and pectin.	4 weeks	+0.15 (*p* = NS) vs. no intervention; −0.07 vs. pomace (*p* = NS); 0.42 vs. cloudy juice (*p* = NS); −0.36 vs. clear juice (*p* = NS).	0.0 WHR vs. no intervention (*p* = NS); 0.003 WHR vs. pomace (*p* = NS); −0.008 WHR vs. cloudy juice (*p* = NS); −0.005 WHR vs. clear juice (*p* = NS).	No power calculation. Not preregistered. Very small sample size for number of groups. High dropout rate.
Agebratt et al. (33)	Healthy non-obese men and women. 30 randomized; 15 fruit; 15 nuts.	Diet supplementation with 7 kcal/kg body weight/d of fruit vs. nuts.	8 weeks	+0.03 kg (*p* = 0.95)	−0.2 cm SAD (*p* = 0.91); −0.08 l VF (*p* = 0.16)	Performed a power calculation but based on a hepatic fat outcome. Preregistered but not for anthropometric outcomes (NCT02227511). Registry page is sparse.
Kumari et al. (34)	Men and women 18–25 years old. 45 randomized; 15 guava with peel; 15 peeled guava; 15 no intervention.	400 g guava with skin per day (group A) vs. 400 g peeled guava per day (group B) vs. no intervention (group C)	6 weeks	−2.0 kg (A vs. C; *p* = NR); −2.6 kg (B vs. C; *p* = NR)	Not reported	No power calculation. Not preregistered.

*Randomized controlled trials reporting the impact of whole, fresh fruit consumption on body weight and adiposity. Weight and adiposity differences represent changes in the fruit intervention group relative to the comparator group. Additional strengths and limitations of study design are listed in the “notes” column. NS, not statistically significant (p > 0.05). NR, not reported; BF, body fat; WC, waist circumference; WHR, waist-to-hip ratio; SAD, sagittal abdominal diameter; VF, visceral fat*.

RCTs reporting the impact of whole, fresh fruit consumption on energy intake were published between 2003 and 2016 and are summarized in Table 2. Of the 5 RCTs identified, two included fewer than 20 subjects, two included 21–50 subjects, and one included more than 50 subjects. Interventions represented several fruit types, but apple (*n* = 2) was named more commonly than other fruit. As in the body weight RCTs (some of which also met inclusion criteria for energy intake), the quantity of fruit varied widely, with the highest intake representing a goal of 15 percent of calorie intake from fruit-derived fructose, which implies ~30 percent of total energy intake from fruit. Interventions lasted between one meal and 12 weeks, with single-meal and 8-week trials being the most common. Three trials used self-report methods to measure energy intake, two of which used a 3-day weighed food record; food intake in the other two trials was directly measured by investigators. The latter two trials were single-meal studies. Three trials reported performing a power calculation as part of study design, although one was powered for a hepatic fat outcome. Only one trial was preregistered, also for a hepatic fat outcome.

**Table 2:** Secondary outcome: impact of whole, fresh fruit consumption on energy intake in RCTs.

**Trial**	**Subjects**	**Intervention**	**Duration**	**Energy intake difference**	**Measurement method**	**Notes**
de Oliveira et al. (26)	Hypercholesterolemic, overweight, non-smoking women, 30 to 50 years of age. 49 randomized; number in each group unclear.	3 apples, pears, or oat cookies per day. Hypocaloric diet for all subjects.	12 weeks	−22 kcal (*p* = NR) daily	Food frequency questionnaire and 3-day dietary record	No power calculation. Not preregistered. No between-group *p*-value provided for EI. May have pooled two fruit groups that were individually randomized.
Rodriguez et al. (27)	Women with obesity. 15 randomized; 8 low-fruit; 7 high-fruit.	Low-fruit diet vs. high-fruit diet (goal of 5 vs. 15% of kcal from fructose). Hypocaloric diet for all subjects.	8 weeks	+47 kcal (*p* = NS) daily	3-day dietary record	No power calculation. Not preregistered. Very small sample size
Flood-Obbagy and Rolls (35)	Men and women 18–45 years old. 59 randomized; crossover design.	Isocaloric preload with apple, apple sauce, apple juice with added fiber, apple juice, or no preload. Followed by an *ad libitum* test meal.	Single meal	−187 kcal vs. no preload (*p* < 0.0001); −91 kcal vs. apple sauce (*p* < 0.02); −152 kcal vs. apple juice with fiber (*p* < 0.02); −178 kcal vs. apple juice (*p* < 0.02). Figures represent total meal energy intake including preload.	Weighed by investigators	Performed a power calculation. Not preregistered.
James et al. (36)	Healthy pre-menopausal women. 12 randomized; crossover design.	Isocaloric mixed berries vs. confectionary snack, followed by an *ad libitum* test meal.	Single meal	−134 kcal (*p* < 0.001)	Weighed by investigators	Performed a power calculation. Not preregistered.
Agebratt et al. (33)	Healthy non-obese men and women. 30 randomized; 15 fruit; 15 nuts.	Diet supplementation with 7 kcal/kg body weight/d of fruit vs. nuts.	8 weeks	−216 kcal (*p* = 0.37)	3-day weighed dietary record	Performed a power calculation but based on hepatic fat outcome. Preregistered but not for energy intake (NCT02227511). Registry page is sparse.

*Randomized controlled trials reporting the impact of whole, fresh fruit consumption on energy intake. Energy intake differences represent the fruit intervention group relative to the comparator group. Additional strengths and limitations of study design are listed in the “notes” column. NS, not statistically significant (p > 0.05); NR, not reported*.

Prospective observational studies reporting the impact of whole, fresh fruit consumption on body weight and adiposity change were published between 2002 and 2018 and are summarized in Table 3. Of the 25 studies identified, seven included fewer than 1,000 subjects, six included 1,000–10,000 subjects, seven included 10,001–50,000 subjects, and five included more than 50,000 subjects. Follow-up length ranged from 6 months to 24 years. Eleven studies reported weight changes, and 18 reported changes in other measures of adiposity. None were preregistered, and only one applied an adjustment for multiple comparisons (Bonferroni correction) to control family-wise error rate when testing several hypotheses using a single data set.

**Table 3:** Secondary outcome: association of whole, fresh fruit intake with changes in measures of adiposity in prospective observational studies.

**Study**	**Subjects**	**Intake measure**	**Follow-up length**	**Weight change**	**Adiposity change**	**Covariates**	**Notes**
Schulz et al. (37)	17,369 German non-smoking men and women	Food frequency questionnaire	2 years	Not reported	Men: OR per 100 g daily intake for small wt gain 1.04 (*p* = NS); OR for large wt gain 0.94 (*p* = NS); OR for small wt loss 1.05 (*p* = NS); OR for large wt loss 1.03 (*p* = NS). Women: OR per 100 g intake for small wt gain, 0.94 (*p* = NS); OR for large wt gain 0.94 (*p* = NS); OR for small wt loss 1.01 (*p* = NS); OR for large wt loss 1.03 (*p* = NS).	Age, initial body weight and height, education, weight history, medication, menopause, life and health contentment, dietary change, physical activity, prevalent diabetes and thyroid disease	Not adjusted for multiple comparisons. Not preregistered.
Field et al. (38)	14,918 US boys and girls ages 9–14	Food frequency questionnaire	3 years	Not reported	Boys: 0.0 SD BMI *z*-score (*p* = NS); Girls: 0.0 SD BMI *z*-score (*p* = NS)	Age, age squared, Tanner stage, height change, baseline weight, physical activity, and inactivity	Not adjusted for multiple comparisons. Not preregistered.
Newby et al. (39)	1,379 US boys and girls ages 2–5	Food frequency questionnaire	6–12 months	+0.02 kg/year per daily serving (*p* = 0.41)	Not reported	Age, sex, ethnicity, residence, level of poverty, maternal education, birth weight, food groups	Not adjusted for multiple comparisons. Not preregistered.
Drapeau et al. (40)	248 Canadian men and women	3-day dietary record	6 years	−0.18 kg per 1 percent increase in fruit intake (*p* = 0.03)	−0.16 % BF per 1 percent increase in fruit intake (*p* = 0.01); −0.19 cm waist circumference per 1 percent increase in fruit intake (*p* = 0.03); −1.05 mm sum of 6 skinfold thicknesses per 1 percent increase in fruit intake (*p* = 0.01)	Age, baseline body weight, or adiposity indicators, changes in daily physical activity level	Not adjusted for multiple comparisons. Not preregistered.
He et al. (41)	74,063 US female health professionals ages 38–63	Food frequency questionnaire	12 years	Not reported	OR for obesity 0.76 in highest vs. lowest quintile of fruit intake change (*p* = 0.0007); OR for major weight gain 0.73 in highest vs. lowest quintile of fruit intake change (*p* = 0.03)	Age, year of follow-up, change in physical activity, change in cigarette smoking status, changes in alcohol consumption and caffeine intake, change in use of hormone replacement therapy, changes in energy-adjusted intakes of saturated fat, polyunsaturated fat, monounsaturated fat, trans-unsaturated fatty acid, protein, total energy, baseline BMI	Not adjusted for multiple comparisons. Not preregistered.
Koh-Banerjee et al. (42)	27,082 US male health professionals ages 40–75	Food frequency questionnaire	8 years	−2.51 kg per 20 g/d increase in fruit fiber (*p* < 0.001)	Not reported	Age, baseline fruit intake, smoking, baseline weight, and baseline values and changes in refined grains, calories, total physical activity, alcohol, protein, and trans, saturated, monounsaturated, and polyunsaturated fats	Not adjusted for multiple comparisons. Not preregistered.
Nooyens et al. (43)	288 Dutch men ages 50–65 years	Food frequency questionnaire	5 years	−0.02 kg/year per serving increase of fruit per week (*p* = 0.03)	−0.03 cm/year WC per serving increase of fruit per week (*p* < 0.01)	Retirement status, type of job, interaction between retirement and type of job, age, smoking, baseline fruit intake, physical activity, intake of potatoes, breakfast, sugar-sweetened beverages, fiber density	Not adjusted for multiple comparisons. Not preregistered.
Sanchez-Villegas et al. (44)	6,319 Spanish male and female university graduates	Food frequency questionnaire	28 months	−0.09 kg in highest vs. lowest tertile of fruit intake (*p* = 0.46)	Not reported	Age, sex, baseline BMI, smoking, physical activity, alcohol consumption, energy, intake, change in dietary habits, physical activity, intake of cereals, vegetables, legumes, fish, nuts, meat, full-fat dairy, olive oil, red wine	Not adjusted for multiple comparisons. Not preregistered.
te Velde et al. (45)	168 Dutch men and women	Dietary history interview	24 years	Not reported	−0.71 BMI units in highest vs. lowest quartile of fruit intake (*p* = NS); −0.16 mm sum of 6 skinfolds in highest vs. lowest quartile of fruit intake (*p* = NS)	Sex, bone age at 13 years, total energy intake, physical activity, tobacco use, fiber intake	Not adjusted for multiple comparisons. Not preregistered.
Vioque et al. (46)	206 Spanish men and women ages 15–80	Food frequency questionnaire	10 years	Not reported	OR 0.62 for weight gain >3.41 kg over 10 years in highest vs. lowest quartile of fruit intake (*p* = 0.211)	Sex, age, educational level, BMI, time spent watching TV, presence of disease, baseline height, energy intake, energy-adjusted intakes of protein, saturated fat, monounsaturated fat, polyunsaturated, fiber, caffeine, alcohol	Not adjusted for multiple comparisons. Not preregistered.
Buijsse et al. (47)	89,432 Danish, German, UK, Italian, and Dutch men and women	Food frequency questionnaire	6.5 years	−0.016 kg/y per additional 100 g fruit intake (*p* < 0.05)	Not reported	Age, sex, cohort, product term UK-Nor X fruit/vegetable intake, years of follow-up, baseline weight, baseline height, change in smoking status, baseline physical activity (dummy variables), education, alcohol intake, postmenopausal status, postmenopausal hormone use	Not adjusted for multiple comparisons. Not preregistered.
Halkjaer et al. (48)	42,696 Danish men and women ages 50–64	Food frequency questionnaire	5 years	Not reported	Men: −0.07 cm WC per additional 60 kcal/d fruit intake (*p* < 0.001). Women: −0.10 cm WC per additional 60 kcal/d fruit intake (*p* < 0.07).	Baseline waist circumference, body mass index, age, smoking, sport, hours of sport, energy intake from wine, beer, and spirits, baseline intake of 21 food and beverage groups	Not adjusted for multiple comparisons. Not preregistered.
Berz et al. (49)	2,327 US girls age 9 years	3-day dietary record	10 years	Not reported	−2.1 BMI units in highest vs. lowest fruit intake (*p* < 0.001)	Race, height, SES, physical activity level, television viewing and video game playing, and total energy	Not adjusted for multiple comparisons. Not preregistered.
Mozaffarian et al. (50)	120,877 US male and female health professionals	Food frequency questionnaire	12–20 years	−0.22 kg per 4-year period per 1-serving increase (*p* < 0.001)	Not reported	Age, baseline BMI, sleep duration, changes in physical activity, alcohol use, television watching, smoking, vegetables, nuts, dairy, potatoes, grains, sugar-sweetened beverages, fruit juice, diet beverages, sweets, meats, trans fat, fried foods	Not adjusted for multiple comparisons. Not preregistered.
Romaguera et al. (51)	48,631 Danish, German, UK, Italian, and Dutch men and women	Food frequency questionnaire	5.5 years	Not reported	−0.04 cm/y WC (adjusted for BMI) per 100 kcal increment of fruit intake (*p* < 0.001)	Energy intake, age, baseline weight, baseline height, baseline WC (adjusted for BMI), smoking, alcohol intake, physical activity, education, follow-up duration, menopausal status, hormone replacement therapy	Not adjusted for multiple comparisons. Not preregistered.
Mirmiran et al. (52)	1,930 Iranian men and women ages 19–70	Food frequency questionnaire	3 years	−0.42 kg in highest vs. lowest quartile of fruit intake (*p* = 0.01)	−0.53 cm WC in highest vs. lowest quartile of fruit intake (*p* = 0.006)	Sex, age at baseline, BMI, education, smoking, physical activity, total energy intake, dietary carbohydrate, fat, protein	Not adjusted for multiple comparisons. Not preregistered.
Vergnaud et al. (53)	373,803 Danish, French, German, Greek, Italian, Dutch, Norwegian, Spanish, Swedish, and UK men and women	Food frequency questionnaire	5 years	Men: −0.001 kg/y per additional 100 g fruit intake (*p* = 0.75). Women: −0.001 kg/y per additional 100 g fruit intake (*p* = 0.20).	Not reported	Age, education, physical activity, change in smoking status, BMI at baseline, follow-up time, energy intake, energy intake from alcohol, plausibility of total energy intake reporting, vegetable intake	Not adjusted for multiple comparisons. Not preregistered. Intake data were calibrated using 24-h dietary recall data.
Bayer et al. (54)	1,252 girls and boys age 6	Parental questionnaire	4 years	Not reported	+0.014 unit BMI z-score in high vs. low fruit consumers (*p* = NS). +0.033 unit BMI *z*-score for decreased fruit intake (*p* = 0.808). −0.126 unit BMI *z*-score for increased fruit intake (*p* = 0.348).	Physical activity, cluster	Not adjusted for multiple comparisons. Not preregistered.
Bertoia et al. (55)	133,468 US male and female health professionals	Food frequency questionnaire	13–14 years (mean)	−0.24 kg per 4-year period per 1-serving increase (*p* < 0.05)	Not reported	Baseline age, BMI, change in smoking status, physical activity, hours of sitting or watching TV, hours of sleep, fried potatoes, juice, whole grains, refined grains, fried foods, nuts, whole-fat dairy, low-fat dairy, sugar-sweetened beverages, sweets, processed meats, non-processed meats, trans fat, alcohol, seafood	Not adjusted for multiple comparisons. Not preregistered. Similar to Mozaffarian et al. (50).
de Munter et al. (56)	23,108 Swedish men and women ages 18–84	Questionnaire	8 years	Not reported	Men: −0.07 BMI units in “≥ daily” vs. “less than daily” fruit intake group (*p* = NS); RR 0.89 for overweight incidence (*p* = NS); RR 0.90 for obesity incidence (*p* = NS). Women: +0.02 BMI units in “≥ daily” vs. “less than daily” fruit intake group (*p* = NS); RR 0.94 for overweight incidence (*p* = NS); RR 0.88 for obesity incidence (*p* = NS).	Age, education, physical activity, alcohol intake, smoking	Not adjusted for multiple comparisons. Not preregistered.
Rautiainen et al. (57)	18,146 US female health professionals aged ≥45	Food frequency questionnaire	15.9 years	−0.01 kg in highest vs. lowest quintile of fruit intake (*p* = 0.46)	HR 0.87 for obesity or overweight in highest vs. lowest quintile of fruit intake (*p* = 0.01)	“Age, randomization treatment assignment, physical activity, history of hypercholesterolemia or hypertension, smoking status, postmenopausal status, postmenopausal hormone use, alcohol use, multivitamin use, energy intake, baseline BMI”	Not adjusted for multiple comparisons. Not preregistered.
Hur et al. (58)	770 Korean male and female children and adolescents	3-day dietary record	4 years	Not reported	−0.08 unit BMI z-score per g/d fruit sugar (*p* < 0.05). −0.60% BF per g/d fruit sugar (*p* = NS).		Not adjusted for multiple comparisons. Not preregistered.
Bel-Serrat et al. (59)	2,755 Irish boys and girls ages 6–10	Parental food frequency questionnaire	3 years	Not reported	OR 2.16 for developing overweight/obesity in “sometimes/never” fresh fruit vs. “every day/most days” group (*p* < 0.01). −0.04 unit BMI *z*-score in “sometimes/never” fresh fruit vs. “every day/most days” group (*p* = NS).	Energy intake, income, sex, age	Not adjusted for multiple comparisons. Not preregistered.
Mumena et al. (60)	336 Caribbean (St. Kitts and Nevis, Trinidad and Tobago) boys and girls ages 6–10	24-h dietary recall	18 months	Not reported	Children who became overweight or obese had a lower fruit intake at baseline than children who did not (*p* = 0.017)	Measurement round, follow-up length, age, sex, baseline *z*-BMI, baseline abdominal obesity status, school socioeconomic status, school location, household ownership	Adjusted for multiple comparisons (Bonferroni). Not preregistered.
Okop et al. (61)	800 South African men and women	Food frequency questionnaire	4.5 years	Not reported	OR 1.47 of weight gain ≥5% in “seldom or no daily fruit” vs. “daily fruit” (*p* < 0.05)	N/A	Not adjusted for multiple comparisons. Not preregistered.

*Prospective observational studies reporting the association between whole, fresh fruit consumption and body weight and adiposity changes. Additional strengths and limitations of study design are listed in the “notes” column. NS, not statistically significant (p > 0.05); NR, not reported; OR, odds ratio; RR, relative risk; HR, hazard ratio; BMI, body mass index; BF, body fat; WC, waist circumference*.

### Risk of Bias

**Figure 2:**
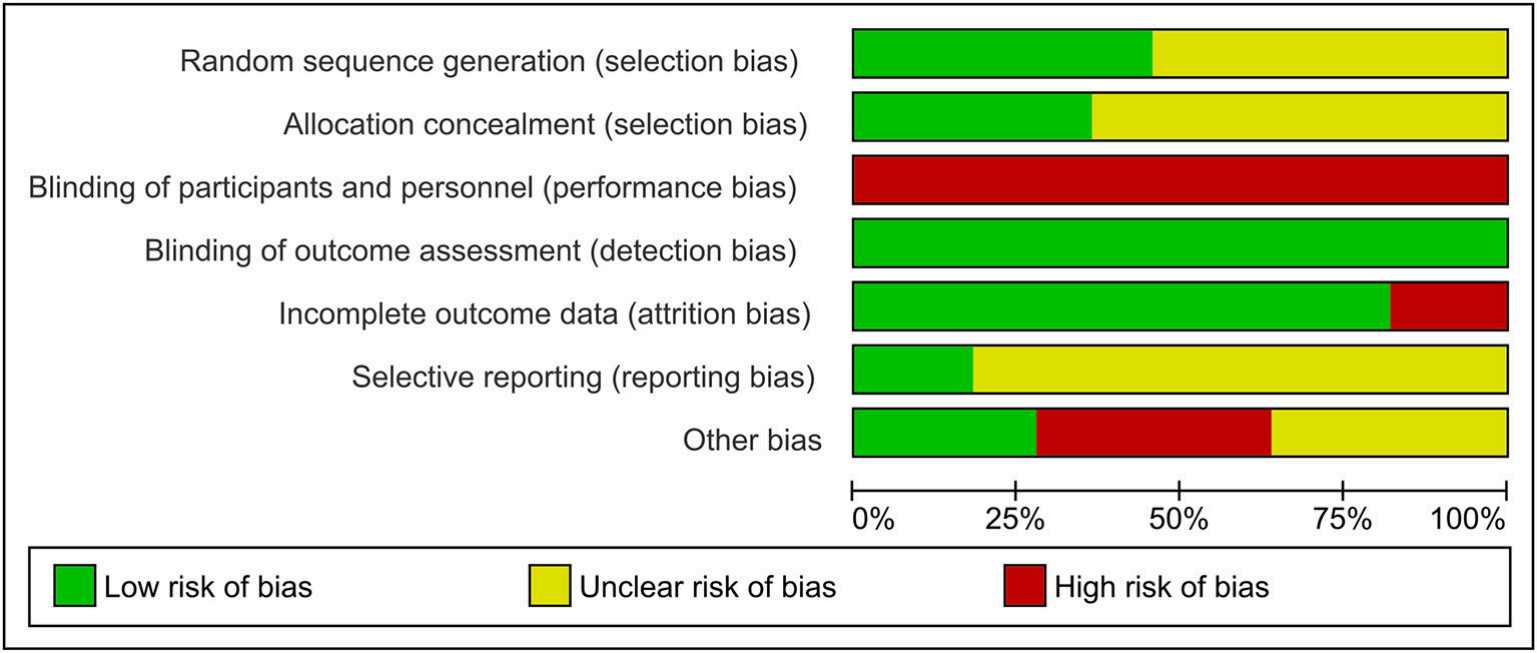
Risk of bias graph for the primary outcome: the impact of whole, fresh fruit consumption on measures of body weight and adiposity in RCTs. Bars illustrate the proportion of trials that received a particular risk of bias score in each risk of bias domain.

**Figure 3:**
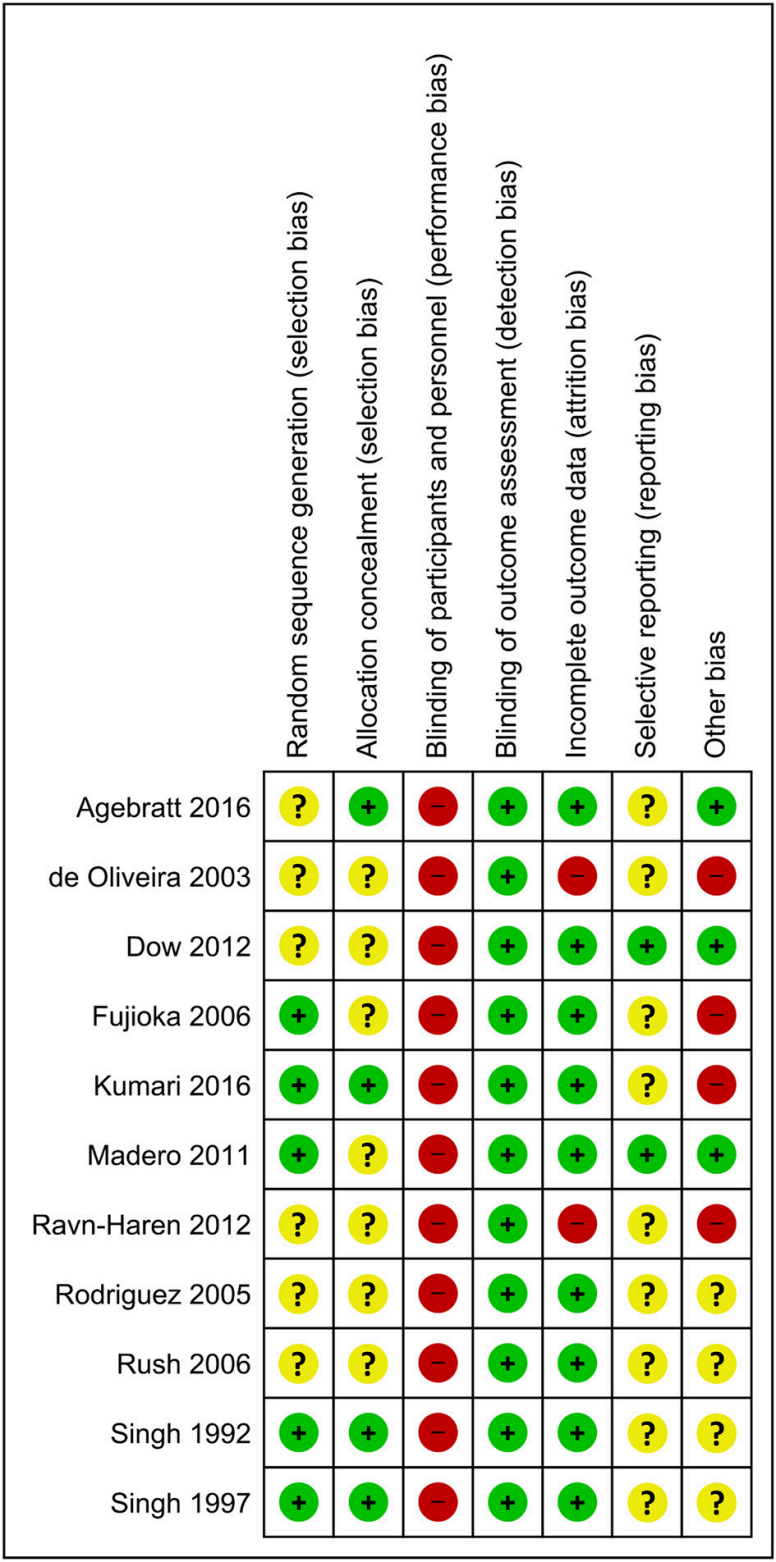
Risk of bias summary for individual trials contributing to the primary outcome: the impact of whole, fresh fruit consumption on measures of body weight and adiposity in RCTs. Colored dots represent low risk (green), unclear risk (yellow), and high risk (red) in each risk of bias domain for each trial.

Figure 2 presents the Cochrane risk of bias graph, and Figure 3 presents the Cochrane risk of bias summary for RCTs reporting the impact of whole, fresh fruit consumption on body weight and other measures of adiposity. Six of 11 included trials are at an unclear risk of bias from random sequence generation and allocation concealment due to incomplete description of the randomization process, while the other five are at a low risk of bias. All 11 trials are at a high risk of bias due to lack of blinding of participants, an unavoidable consequence of including whole, fresh fruit consumption as an intervention. This leaves open the possibility that part of the impact of fruit observed in these trials is attributable to placebo effects. The risk of bias due to blinding of outcome assessment is low in all 11 trials, not because investigators were consistently blinded to treatment assignment, but because there is a low risk of bias in measuring body weight due to its simple and objective nature. Nine of 11 trials had a low risk of attrition bias due to low dropout rates, while the other two were at high risk due to high dropout rates. Nine of 11 trials were at an unclear risk of selective reporting bias due to insufficient information about initial study design resulting from a lack of detailed preregistration information, while the other two were at low risk due to detailed preregistration. The risk of other bias was fairly evenly split between low (*n* = 3), unclear (*n* = 4), and high (*n* = 4) risk of bias. The two studies with a low risk of other bias were scored as such because they offered detailed preregistration information with primary outcomes relevant to adiposity.

Body weight RCTs that scored most favorably on the Cochrane risk of bias tool are Madero et al. (30), Dow et al. (31), Agebratt et al. (33), Singh (25), Singh et al. (24), and Kumari et al. (34) (Figure 3). Each received a score of “low risk” in four or five of seven domains.

**Figure 4:**
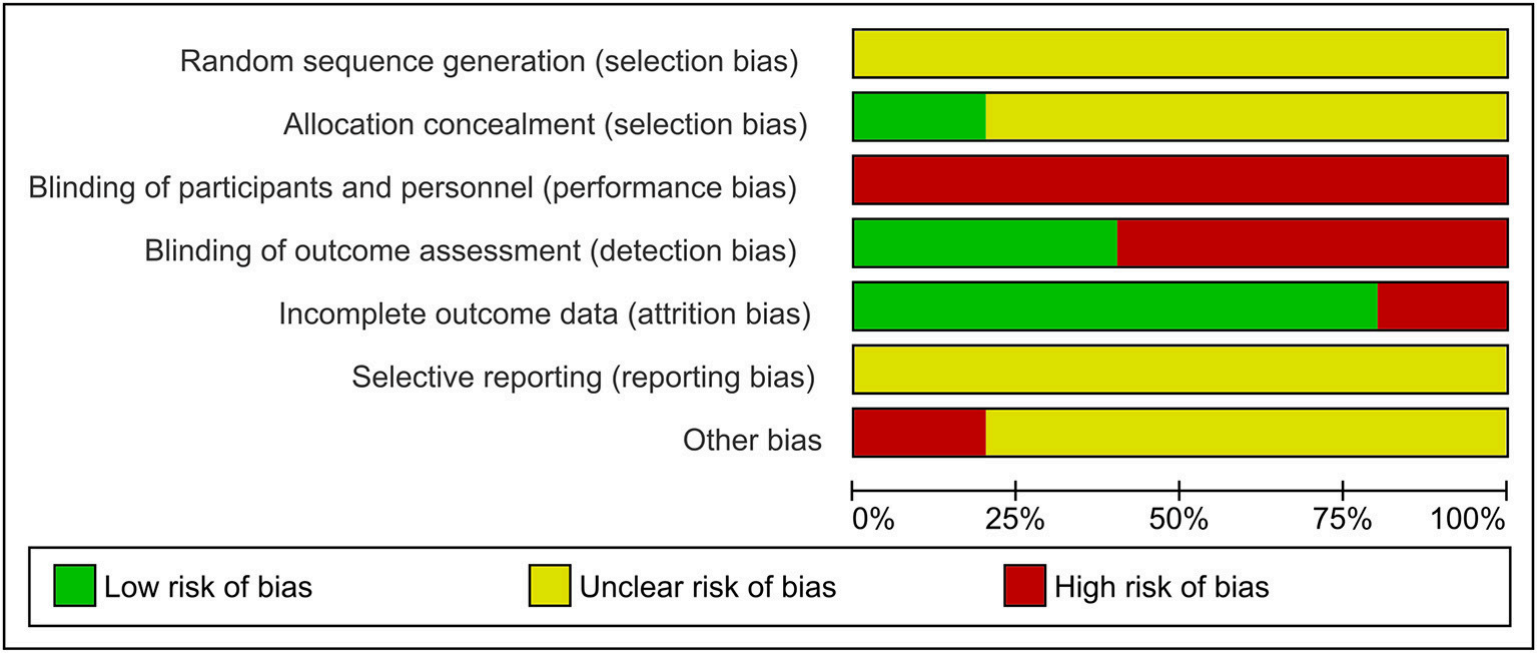
Risk of bias graph for a secondary outcome: the impact of whole, fresh fruit consumption on energy intake in RCTs. Bars illustrate the proportion of trials that received a particular risk of bias score in each risk of bias domain.

**Figure 5:**
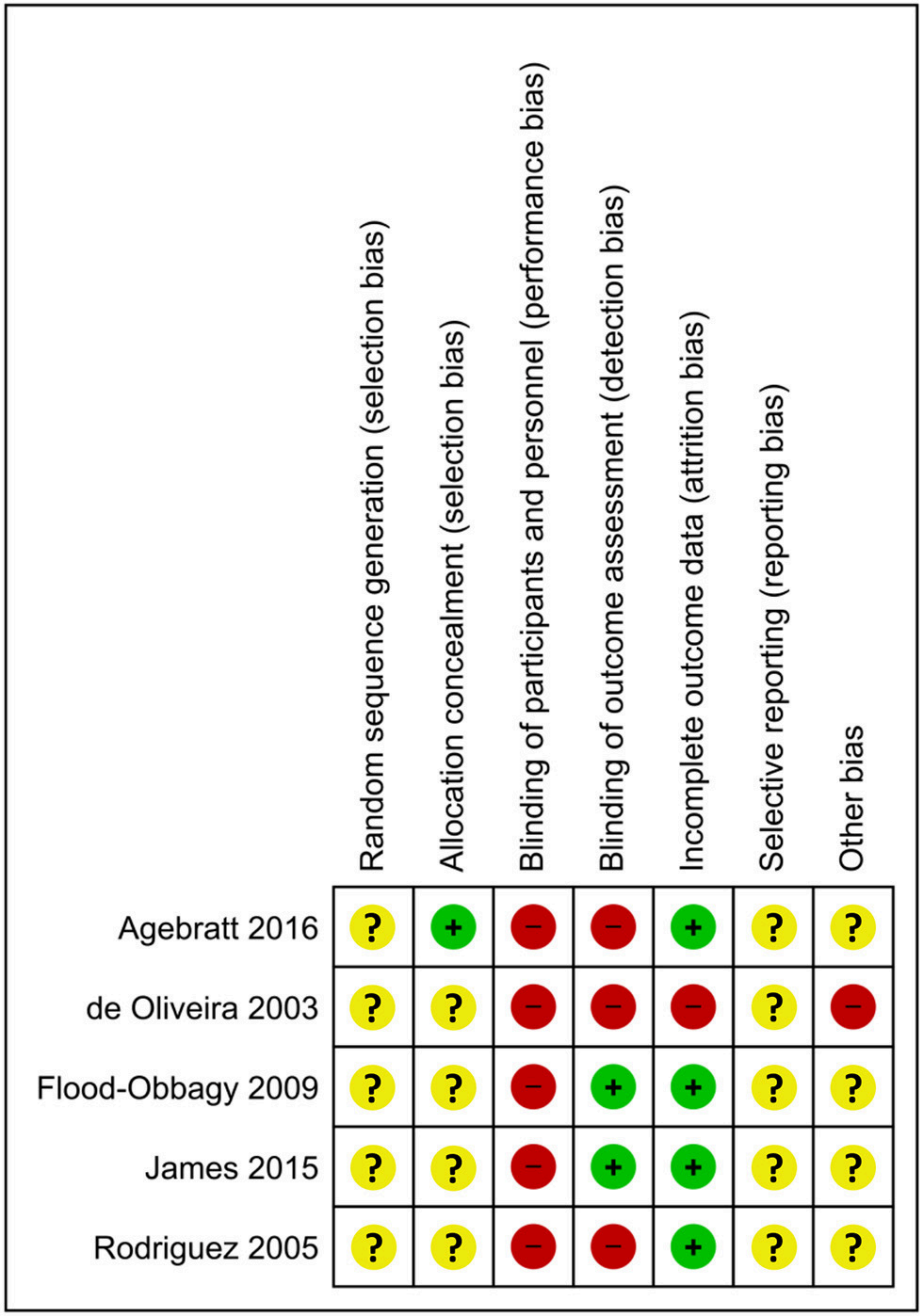
Risk of bias summary for individual trials contributing to a secondary outcome: the impact of whole, fresh fruit consumption on energy intake in RCTs. Colored dots represent low risk (green), unclear risk (yellow), and high risk (red) in each risk of bias domain for each trial.

Figure 4 presents the Cochrane risk of bias graph, and Figure 5 presents the Cochrane risk of bias summary for RCTs reporting the impact of whole, fresh fruit consumption on energy intake. This literature fared more poorly in risk of bias scoring than the body weight RCT literature. All energy intake RCTs are at an unclear risk of bias from random sequence generation, and four of five are at an unclear risk of bias from allocation concealment. This is due to incomplete description of the randomization process. For the same reason as the body weight RCTs, all energy intake RCTs are at a high risk of bias due to lack of blinding of participants, leaving open the possibility of placebo effects. The risk of bias due to blinding of outcome assessment is high in three of five energy intake RCTs due to reliance on self-reported measures of energy intake, and low in two of five due to directly measured energy intake. Four of five trials had a low risk of attrition bias due to low dropout rates. All trials were at an unclear risk of selective reporting bias due to insufficient information about initial study design resulting from a lack of detailed preregistration information. The risk of other bias was unclear in four of five trials due to insufficient information.

Energy intake RCTs that scored most favorably on the Cochrane risk of bias tool are Flood-Obbagy and Rolls (35), James et al. (36), and Agebratt et al. (33) (Figure 5), although none received a score of “low risk” in more than two of seven domains.

Although observational studies were not assessed using the Cochrane risk of bias tool, features that are informative of bias risk will nevertheless be considered here. All observational studies included in this review used self-report methods to measure fruit intake, most commonly food frequency questionnaires (Table 3). This introduces a substantial source of error that may also introduce an unknown degree of bias. Validation studies suggest that the Pearson correlation coefficient between fruit intake measured by food frequency questionnaires and 7-day weighed food record is 0.50–0.67, implying that 65–75 percent (*R*^2^) of the variability in fruit intake identified by 7-day weighed food record is not captured by food frequency questionnaire (62). Although it has been argued that self-report error is randomly distributed and should not bias associations, the author is uncertain to what extent this argument is correct, and in which contexts.

Observational studies are inherently more limited than RCTs as tools for causal inference because relationships between exposure and outcome variables may be confounded by other variables. For example, between-person variation in fruit and vegetable intake has a genetic component and “individuals genetically predisposed to low fruit and vegetable consumption may be predisposed to higher [body mass index],” suggesting a possible source of bias that could be both important and difficult to correct (63). For this reason among others, observational relationships between fruit intake and body weight changes can be difficult to interpret. Most of the studies represented in Table 3 adjusted extensively for confounding variables in an attempt to limit confounding bias. However, it is difficult to be certain that the most impactful confounding variables were measured and appropriately incorporated into multivariate models.

In addition, it is difficult to be certain that observational relationships were not overadjusted, attenuating the measured association between fruit intake and body weight/adiposity outcomes. In this regard, it is notable that many studies adjusted for energy intake, which seems suboptimal for the purposes of this review since modified energy intake may be a key intermediate variable between fruit intake and body weight/adiposity outcomes.

An additional potential source of bias is that none of the observational studies reported preregistration, leaving open the possibility that outcome selection and data analysis methods were (perhaps inadvertently) guided toward preferred outcomes. The great diversity of analytic methods represented in these studies amplifies this concern because it demonstrates that the possible space of analytic methods is vast (64). Preregistration narrows this space *a priori*.

Finally, only one observational study reported adjusting significance tests for multiple comparisons, e.g., Bonferroni correction. The more hypothesis tests that are performed on a single data set, the higher the likelihood of a false positive finding. Since the commonly accepted false positive rate in the biomedical research community is 5 percent, testing ten hypotheses yields a 40 percent risk of that at least one of the ten tests will return a false positive finding. Many of the observational studies identified here tested ten or more hypotheses, and most represent datasets that have been analyzed using many statistical tests in other contexts (64). Overall, the risk of bias in the observational literature considered here appears quite high and must limit the strength of causal inferences drawn from it.

### Synthesized Findings

#### Body Weight RCTs

The primary outcome of this review is the impact of whole, fresh fruit consumption on measures of adiposity including body weight, as measured by RCTs. Given the limited number of studies identified, which would be further reduced by more stringent meta-analysis inclusion criteria, and large variation of methodology and study quality, qualitative synthesis appears most appropriate.

Of the 11 RCTs included, seven reported numerical reductions of body weight as a result of increased fruit consumption relative to a comparison group, while four reported numerical increases of weight (Table 1). Among trials that reported numerical reductions of body weight, three comparisons were statistically significant (26, 28, 30), while none of the comparisons suggesting weight increases achieved statistical significance. This is consistent with the possibility that the weight increases result from random chance. In support of this possibility, studies that reported non-significant weight increases from fruit consumption tended to be statistically underpowered, with as few as six subjects per group (Table 1).

Among the two trials that reported performing a power calculation *a priori* and were preregistered with measures of adiposity as the primary outcome, Madero et al. (30) reported a significant weight loss of −1.36 kg over 6 weeks of substantially increased fruit consumption (30), while Dow et al. (31) reported a non-significant weight loss of −0.5 kg over 6 weeks of modestly increased fruit consumption (31). In addition, Madero et al. (30) received the lowest risk of bias score of all studies considered, and Dow et al. (31) tied for the second lowest risk of bias. In contrast, three of the four trials reporting non-significant increases of body weight from increased fruit consumption were among those with the highest risk of bias, while the fourth, which reported a non-significant and negligible weight gain of 0.03 kg over 8 weeks, was among those with relatively lower risk of bias (Figure 3). It is evident that the trials reporting weight reductions tend to have higher methodological quality than those reporting weight increases, suggesting that the former should be viewed as more informative.

Six trials reported measures of adiposity other than body weight, including body fat percentage, waist circumference, waist-to-hip ratio, sagittal abdominal diameter, and visceral fat volume (Table 1). Only one measure achieved statistical significance, a waist circumference reduction of −3.1 cm over 8 weeks of a high-fruit diet (27). However, this finding is questionable due to the study's small sample size, high risk of bias, and apparent lack of correction for multiple comparisons.

Since Madero et al. (30) and Dow et al. (31) appear to surpass the others in methodological quality and relevance to adiposity modification, these will be discussed in detail and will contribute disproportionately to the overall conclusions of this review (30, 31). These two trials are particularly relevant to adiposity modification because all subjects had overweight or obesity. Madero et al. (30) was preregistered with anthropometric changes as the primary outcome, and sample size was selected using a power calculation. Sample size was larger than all but one other trial, which had the same number of subjects. This trial also received the lowest risk of bias score among the 11 trials identified, with low risk in all categories except participant blinding, in which high risk is unavoidable due to the impossibility of blinding subjects, and allocation concealment, which was judged as unclear because it was not described in the manuscript.

One hundred and thirty one men and women with overweight or obesity were randomly assigned to eat a low-fructose diet (<20 g/d) vs. a natural-fructose diet (50–70 g/d) in which most of the fructose was from whole, fresh fruit, for 6 weeks. The latter is approximately equivalent to five to eight whole medium apples per day, or eleven to fifteen whole oranges per day. After 6 weeks, the natural-fructose group had lost 1.36 kg more weight than the low-fructose group (*p* = 0.02). The trial also reported non-significant reductions of body fat percentage and waist-to-hip ratio in the natural-fructose group relative to the low-fructose group.

Dow et al. (31) was preregistered with body weight change as the primary outcome, and sample size was selected using a power calculation (31). This trial tied for the second-lowest risk of bias score among the 11 trials identified, with low risk of bias in four of seven domains (Figure 3). Its risk of bias in participant blinding was judged as high, which is unavoidable. Its risk of bias due to randomization and allocation concealment are unclear due to a lack of information in the manuscript.

Seventy-four men and premenopausal women with overweight or obesity were randomly assigned to eat half a fresh grapefruit three times per day prior to each meal, vs. no intervention, for 6 weeks. In addition, all subjects were assigned to a baseline diet low in fruit and vegetables. After 6 weeks, the grapefruit group had lost 0.5 kg more weight than the control group, but this difference was not statistically significant (*p* = 0.119). Between-group differences in body fat percentage (+0.55 percent), waist circumference (−1.22 cm), and waist-to-hip ratio (0.0) were also non-significant.

Informally weighting the strength of findings according to study quality and risk of bias, the overall RCT literature suggests that increasing whole, fresh fruit consumption promotes weight maintenance or modest weight loss over periods of 3–24 weeks, with limited evidence suggesting that high intakes of fruit lead to weight loss among people with overweight or obesity. The overall quality of evidence according to the GRADE method is moderate, indicating that the true effect of fruit consumption on measures of adiposity is probably close to the effect estimated in the RCTs considered here, particularly those of higher quality.

### Energy Intake RCTs

A secondary outcome of this review is the impact of whole, fresh fruit consumption on measures of energy intake, as measured by RCTs. Given the small number of studies identified, and large variation of methodology and study quality, qualitative synthesis appears most appropriate.

Of the five RCTs included, four reported numerical reductions of energy intake as a result of increased fruit consumption relative to a comparison group, while one reported a numerical increase of energy intake (Table 2). Among trials that reported numerical reductions of energy intake, two comparisons were statistically significant (35, 36), while the comparison suggesting numerically increased energy intake reported a small-magnitude effect (+47 kcal/d) that did not achieve statistical significance (27). This is consistent with the possibility that the finding of increased energy intake resulted from random chance. In support of this possibility, this study was likely statistically underpowered, with seven and eight subjects per group (Table 1). It did not report performing an *a priori* power calculation and was also at high risk of bias due to its use of a self-reported measure of energy intake, and not blinding subjects (unavoidable) or investigators (avoidable) (Figure 5).

Only one energy intake trial was preregistered, and its primary outcome was a change of hepatic fat content rather than energy intake (33). Among the three trials that reported performing a power calculation *a priori*, two reported statistically significant single-meal reductions of energy intake of 134–187 kcal from meals including a fruit preload relative to no preload or a confectionary snack (35, 36), and the third reported a non-significant 216-kcal reduction of daily energy intake from a high-fruit diet relative to a high-nut diet (33). The former two trials were the only two to employ direct measurement of energy intake by investigators rather than self-reported intake (Table 2). The three trials that performed power calculations received the most favorable risk of bias scores among the five trials identified, although none of the five trials received a low risk score in more than two of seven domains (Figure 5).

Consistent with the adiposity RCTs, it is evident that the trials reporting energy intake reductions, and particularly statistically significant ones, tend to have higher methodological quality than the trial reporting energy intake increase, suggesting that the former should be viewed as more informative. However, it is notable that the three trials reporting non-significant effects, while relying on self-reported data, were the only three with follow-up periods longer than a single meal (Table 2).

Flood-Obbagy and Rolls (35) and James et al. (36) appear to surpass the other trials in methodological quality due to direct measurement of energy intake, lower risk of bias score than the other three, and sufficient statistical power supported by *a priori* power calculations (35, 36). Flood-Obbagy and Rolls (35) also has the largest sample size of the five trials identified, and its effective sample size is amplified by its crossover design. These two trials will be discussed in detail and will contribute disproportionately to the overall conclusions of this review. Although these trials may provide a higher level of certainty than the others, they are less relevant to energy intake control in the context of obesity because their subjects were either lean (35) or mildly overweight (36) on average.

Flood-Obbagy and Rolls (35) received one of the lowest risk of bias scores among the five trials identified, although none of the trials were at a low risk of bias. It received a low risk of bias score in blinding of outcome assessment due to directly measured energy intake, and low risk of attrition bias due to low attrition (Figure 5). It received an unclear risk of bias score for random sequence generation, allocation concealment, selective reporting, and other bias due to insufficient information in the manuscript and the absence of preregistration. It received a high risk of bias score in participant blinding, in which high risk is unavoidable.

Fifty-nine men and women 18–45 years old, with body mass index of 23.7 kg/m^2^ (M) and 24.3 kg/m^2^ (W), received five food preloads in random order on different days, followed after 15 min by an *ad libitum* test meal of cheese tortellini, tomato sauce, and water (35). Preload conditions were whole apple, apple sauce, apple juice with added soluble fiber (pectin), apple juice, or no preload. All preloads were adjusted to contain equal energy (125 kcal) and weight (266 g) except the no-preload control. Total meal energy intake as directly measured by investigators, including preload, was lowest in the whole apple condition and highest in the no-preload condition, with a highly statistically significant difference of −187 kcal (Table 2). Energy intake in the whole apple condition was also significantly lower than all other conditions. Total meal energy intake increased with each processing step away from whole fresh apples, in the following order: whole apples < apple sauce < apple juice with fiber < apple juice.

James et al. (36) received a risk of bias score identical to Flood-Obbagy and Rolls (35) for similar reasons (Figure 5) (36). Twelve healthy pre-menopausal women, with body mass index of 26.6 kg/m^2^, received two preloads in random order on different days, followed after 60 min by an *ad libitum* test meal of pasta with Bolognese sauce and olive oil. Preload conditions were fresh mixed berries vs. soft berry-flavored candies and were matched for energy content (65 kcal). The two preloads also contained a similar amount of sugar (12.1 vs. 15.5 g). Energy intake at the test meal, as directly measured by investigators, was 134 kcal lower in the mixed berry condition than in the candy condition (*p* < 0.001).

Informally weighting the strength of findings according to study quality and risk of bias, the overall RCT literature suggests that increasing whole, fresh fruit consumption reduces energy intake, particularly when consumed prior to a meal or instead of more energy-dense foods. However, these findings have uncertain relevance to energy intake control in obesity because the most informative trials were conducted in subjects who were lean or modesty overweight. In addition, the most informative trials used single-meal designs, limiting conclusions about the impact of fruit consumption on long-term energy intake. The overall quality of evidence according to the GRADE method is moderate, indicating that the true effect of pre-meal fruit consumption on short-term energy intake in people without obesity is probably close to the effect estimated in the RCTs considered here. Longer-term effects, and those in people with obesity, are less certain. Nevertheless, the energy intake RCT literature is broadly consistent with findings from the body weight RCT literature.

### Prospective Observational Studies

A secondary outcome of this review is the association between whole, fresh fruit consumption and measures of adiposity including body weight, as measured by prospective observational studies. Of the 25 studies identified, 11 reported weight changes over time, and of those, ten reported that people who consumed larger amounts of fruit gained numerically less weight over time (or lost more weight) than people who consumed less fruit (Table 3). Seven of these associations were statistically significant, all suggesting that higher fruit intake is associated with superior weight control over time.

Eighteen studies reported changes in measures of adiposity other than weight over time, 12 of which reported statistically significant differences between higher and lower consumers of fruit (Table 3). Among these 12, all reported that markers of adiposity in people who consume larger amounts of fruit tend to increase less over time (or decline more rapidly) than in people who consume less fruit. Although the Cochrane risk of bias tool was not applied to these studies, as discussed previously they all appear to be at a high risk of bias due to a combination of unavoidable and potentially avoidable design features.

Nevertheless, some studies appear more informative than others. Bertoia et al. (55) [similar to Mozaffarian et al. (50)] and Vergnaud et al. (53) will be discussed further due to the fact that they have the largest sample sizes of the 11 studies identified, they rely on contextually-validated food frequency questionnaires, they have relatively long follow-up periods, and together they represent men and women of 11 nations (35, 36).

Bertoia et al. (55) compiled data from three cohorts representing 133,468 US male and female health professionals (55). Diet assessment was performed using food frequency questionnaires administered at 4-year intervals for a mean of 13–14 years. Analyses controlled for a wide variety of diet and lifestyle factors (Table 3), and although they did not control for demographic variables such as income and education, included cohorts were fairly homogeneous in these respects. Notably, analyses did not control for energy intake, which is preferable because energy intake is likely a mediating variable between whole, fresh fruit intake and adiposity.

In contrast to most other studies identified, Bertoia et al. (55) examined the association between *changes* in self-reported fruit intake and *changes* in measures of adiposity. In other words, if a person reported increasing fruit intake over the course of the follow-up period, were they also less likely to gain weight over time? Although this “change-on-change” method remains fundamentally observational, it may avoid some of the confounding potential of traditional nutritional epidemiology study designs (65). The study reported that a one-serving increase of daily fruit intake was associated with a highly statistically significant 0.24 kg reduction of body weight per 4-year period, and did not report associations with other measures of adiposity.

Vergnaud et al. (53) represents 373,803 Danish, French, German, Greek, Italian, Dutch, Norwegian, Spanish, Swedish, and UK men and women, making it the largest cohort of the 11 studies identified (53). Diet assessment was performed using food frequency questionnaires and the duration of follow-up was 5 years. In contrast to Bertoia et al. (55) but similar to most other nutritional epidemiology studies, Vergnaud et al. (53) reports the association between baseline self-reported fruit intake and weight change over a 5-year period. Analyses controlled for several basic diet, lifestyle, and demographic factors, including energy intake (Table 3). Systematic underestimation or overestimation of dietary intakes between study centers was addressed using a dietary calibration study. The study reported that 100 g higher daily fruit intake was associated with a non-significantly lower rate of weight gain of −0.001 kg per year in men and women, and did not report associations with other measures of adiposity.

Informally weighting the strength of findings according to study quality, the overall prospective observational literature suggests that habitually higher fruit intake is associated with no effect on weight, or modest protection against weight gain. Although these findings must be interpreted cautiously due to limitations of study design, they are broadly consistent with the findings of energy intake and body weight RCTs discussed previously and may suggest that the short- to medium-term effects observed in RCTs persist in the long term.

## Discussion

### Summary of Main Findings

The primary outcome of this review is the impact of whole, fresh fruit consumption on measures of adiposity including body weight, as measured by RCTs. Overall, these RCTs suggest that increasing intake of whole, fresh fruit promotes weight maintenance or modest weight loss over periods of 3–24 weeks. High intakes of whole, fresh fruit in people with obesity may promote some degree of weight loss. RCTs provide little information about more direct measures of adiposity such as body fat percentage. The strength of evidence supporting this conclusion is moderate, indicating that the true effect of fruit consumption on measures of adiposity is probably close to the effect estimated here.

Secondary outcomes of RCTs reporting the impact of fruit consumption on energy intake, and prospective observational studies reporting associations between fruit intake and measures of adiposity, were broadly consistent with the primary outcome. The strength of evidence supporting conclusions regarding energy intake RCTs is moderate, while the prospective observational findings did not receive a GRADE assessment but are likely at high risk of bias. As with the primary outcome, RCTs and prospective observational studies of higher quality tend to support the hypothesis that higher intakes of whole, fresh fruit either do not impact weight or modestly attenuate weight gain over time.

### Limitations

Limitations of this review relate both to the review itself, and to the studies that underlie it. Although quantitative pooling of RCT data appeared suboptimal in this context due to the limited number of studies and considerable heterogeneity in study methods and quality, narrative synthesis is inherently more subjective than quantitative meta-analysis. The author endeavored to limit the potential for bias by preregistering a detailed research plan and adhering to widely accepted defined methods for assessing and reporting evidence, such as the Cochrane risk of bias tool, the GRADE method, and PRISMA guidelines. The author attempted to be transparent in methods and reasoning so the reader may form his or her own views. In addition, the greater subjectivity of narrative reviews may be counterbalanced in some instances by a superior ability to focus on high-quality studies rather than diluting their evidence value by pooling them with lower-quality studies. Finally, the author was not funded for this work and has no connection with Big Fruit, eliminating this potential source of real or perceived bias.

An additional limitation of this review is that due to resource constraints, study selection, data extraction, risk of bias scoring, and GRADE evaluation were performed by one person. The Cochrane handbook for Systematic Reviews of Interventions recommends that systematic reviews be conducted by at least two people to reduce the risk of errors^4^.

The conclusions of this review are also limited by the underlying evidence. Although 11 RCTs were available for the primary outcome of adiposity, most had serious limitations of sample size, lack of preregistration, and/or risk of bias. Conclusions of the primary outcome of this review rest disproportionately on two high-quality trials. Energy intake RCTs were fewer in number and tended to be lower-quality than adiposity RCTs. Prospective observational studies typically had serious limitations including lack of preregistration, lack of correction for multiple comparisons, and potential confounding and overcorrection, which together raise substantial concerns of bias. Some of these limitations are inherent to observational methods, while others are potentially avoidable. Nevertheless, the consistency of findings across the three primary and secondary outcomes is somewhat reassuring.

## Conclusions

Consistent with earlier reviews on this topic (14, 17), available evidence suggests that increasing intake of whole, fresh fruit probably does not increase adiposity, but instead has either no impact on adiposity or constrains it modestly. Findings consistent with this hypothesis are observed in studies representing single meals, 3–24 week periods, and periods of five or more years. Although some uncertainty remains, these findings support increasing the consumption of whole, fresh fruit as part of a multi-factor approach to controlling excess energy intake and adiposity. These findings also suggest that if the sugar content of fruit favors increased energy intake and adiposity, this is outweighed by its other properties, such as lower calorie density, moderate palatability/reward value, higher fiber content, and micronutrient content, at least when consumed as part of typical diet patterns.

These findings support existing recommendations by organization such as the US Department of Agriculture and the World Health Organization to increase fruit consumption as a public health measure (66, 67). Although increasing consumption of whole, fresh fruit is unlikely to have a large impact on population obesity rates on its own, it may make a positive contribution as part of a broader public health strategy for obesity control. Similarly, healthcare providers should not expect large changes in adiposity as a result of increasing whole, fresh fruit consumption alone, but it is reasonable to include it as part of a broader package of slimming diet and lifestyle behaviors. Furthermore, it is unlikely to cause adiposity gain despite its sugar content, at least as part of a typical mixed diet. Increasing intake of whole, fresh fruit may be more effective as an adiposity control measure when presented as a replacement for calorie-dense dessert foods.

Several opportunities for reducing the uncertainty of conclusions on this topic are apparent. Additional high-quality RCTs with changes in adiposity as the preregistered primary endpoint would be useful, particularly if they report an accurate measure of total and regional adiposity such as dual-energy X-ray absorptiometry. High-quality energy intake RCTs, preregistered and with complete description of randomization processes, involving direct measurement of energy intake in people with obesity over longer periods of time would also contribute substantially. Additional energy intake RCTs could also compare the impact of fruit intake in different contexts, such as pre-meal vs. intra-meal vs. after-meal intake, to identify the most effective strategy for energy intake control. Finally, prospective observational studies that use accurate measurement instruments, are conducted according to a preregistered research plan, and adjust for multiple comparisons would reduce uncertainty.

## Author Contributions

SG is the sole contributor to this manuscript, aside from its search strategy, which was designed in collaboration with Ben Harnke.

### Conflict of Interest Statement

This research was not conducted in association with any public or private organization. SG is the author of a general-audience book on the neurobiology of overeating, The Hungry Brain, which does not currently yield royalties but may in the future. This book mentions fruit but does not place a special emphasis on it. SG is the co-creator of a weight management program, the Ideal Weight Program, from which he receives revenue. This program permits the consumption of fruit but does not place a special emphasis on it.

## Footnotes

^1^https://www.crd.york.ac.uk/PROSPERO/display_record.php?RecordID=111830

^2^https://handbook-5-1.cochrane.org/chapter_8/8_assessing_risk_of_bias_in_included_studies.htm

^3^https://handbook-5-1.cochrane.org/chapter_12/12_2_1_the_grade_approach.htm.

^4^https://handbook-5-1.cochrane.org/chapter_2/2_3_4_1_the_importance_of_a_team.htm
